# How Did Human Rights Fare in Amendments to the International Health Regulations?

**DOI:** 10.1017/jme.2024.172

**Published:** 2024

**Authors:** Lisa Forman, Judith Bueno de Mesquita, Luciano Bottini Filho, Benjamin Mason Meier, Matiangai Sirleaf

**Affiliations:** 1: UNIVERSITY OF TORONTO; 2: UNIVERSITY OF ESSEX; 3: SHEFFIELD HALLAM UNIVERSITY; 4: UNIVERSITY OF NORTH CAROLINA AT CHAPEL HILL; 5: O’NEILL INSTITUTE FOR NATIONAL AND GLOBAL HEALTH LAW; 6: UNIVERSITY OF MARYLAND

**Keywords:** International Health Regulations, Human Rights, Equity, Pandemics, Global Health, Governance, COVID-19

## Abstract

In this article, we examine the relationship between the World Health Organization International Health Regulations (IHR) and human rights and its implications for IHR reform, considering the evolution of human rights in the 2005 IHR, the role of human rights in IHR reforms and the implications of these reforms in key domains including equity and solidarity, medical countermeasures, core capacities, travel restrictions, vaccine certificates, social measures, accountability, and financing.

## Introduction

The World Health Organization’s (WHO) International Health Regulations (IHR) are the primary instrument in international law to govern domestic and global responses to public health emergencies. Indeed, the IHR were adopted with the explicit purpose “to prevent, protect against, control and provide a public health response to the international spread of disease.”^1^ Prior to the 2024 amendments, the last revision of the IHR was adopted in 2005, came into effect in 2007, and is automatically binding on all 194 WHO member states. The IHR place a range of obligations on states regarding epidemic and pandemic prevention, preparedness, and response, including to develop minimum core public health capacities to respond to public health emergencies of international concern (PHEIC), notify WHO of events that may constitute a PHEIC, and authorize WHO to issue temporary recommendations during a PHEIC.^2^ Yet the IHR are not the exclusive source of state duties during a PHEIC, with states also bound by interlinked obligations under international human rights law to respect, protect, and fulfill human rights, including the rights to health, life, the benefits of scientific progress, and equality and non-discrimination. These normative and legal overlaps with human rights are formalized in IHR (2005) Article 3, which requires its implementation to be with “full respect for the dignity, human rights and fundamental freedoms of persons.”

Despite these interconnections with human rights, the IHR has historically been more closely linked to the dominant global health security paradigm in global health, which has focused on preventing the spread of infectious diseases across borders, and is attentive not simply to emerging infectious disease threats but also to more traditional national security concerns, including chemical, biological and radio-nuclear threats (CBRN).^3^ Similarly, reflecting the dominance of trade and commercial objectives, the IHR regime’s public health purpose is explicitly adjoined to ensuring that public health regulations do not unduly interfere with international trade.^4^ While the IHR does include explicit and implicit human rights-related provisions, the instrument remains primarily focused on global health security rather than on norms under international human rights law. This focus is a profound mistake given how inextricably a public health response to the international spread of disease is linked to the protection of human rights.^5^ Indeed, the prioritization of global health security and international trade paradigms holds the potential to conflict with both the IHR’s public health objectives and with states’ human rights obligations during a pandemic.

While the IHR does include explicit and implicit human rights-related provisions, the instrument remains primarily focused on global health security rather than on norms under international human rights law. This focus is a profound mistake given how inextricably a public health response to the international spread of disease is linked to the protection of human rights.

The shortcomings of states’ responses to COVID-19 underscored profound gaps and weaknesses in the IHR’s ability to effectively govern a public health response to the international spread of disease while protecting and promoting human rights. States failed to realize the IHR’s purpose of preventing, protecting against, and controlling the emergence and international spread of COVID-19, exposing the weaknesses in domestic health and social security systems as well as in international cooperation under global health governance. More gravely, state implementation of the IHR often entailed neglect as well as serious and widespread violations of a range of human rights, including when it came to social measures, travel restrictions, and equitable access to medical countermeasures. These weaknesses and violations underscored the need to extensively amend the IHR to make it fit for purpose, including through creating more effective accountability mechanisms and defining a broader scope for its operation to embrace human rights, pandemic cycles, health systems, access to pandemic health products like vaccines, and the social determinants of health. It is no surprise in this context that reform of the IHR and the related development of a new WHO pandemic agreement emerged as primary international political responses to resolving the governance gaps illuminated during COVID-19.

As a result, in 2024 the IHR was amended with novel inclusions on equity, solidarity, access to vaccines, financing, and pandemic preparedness. While these amendments may mitigate some of the IHR’s negative human rights impacts, they are vague and have done little to augment the IHR’s most contentious provisions (such as travel restrictions). More concerningly, the amendments are not explicitly connected to human rights in key domains including vaccine certificates, social measures, inequitable access to medical countermeasures, and inadequate development of core health care and system capacities.

We argue that what are required instead are clearer and more explicit and direct normative and operational synergies and linkages to international human rights law and institutions. These synergies and linkages are not simply necessary for states to better fulfill their international human rights obligations during future PHEICs but are essential to the success of national and global governance of future epidemic and pandemic disease threats. Greater attention to human rights in the IHR would better balance global health security and trade imperatives with public health and human rights obligations during a PHEIC. More broadly, these linkages are important to ensure that the IHR follows the principle of systemic integration within international law, in which the functional areas of global health law — including human rights law — are linked to each other rather than further fragmented in ways that are harmful to the global public good.

In this article we analyze the relationship between the 2005 IHR as amended and human rights in the following ways. We begin by examining the evolution and efficacy of human rights leading into the 2005 revision of the IHR. Analyzing the challenges of COVID-19 and the 2024 amendments of the IHR, we then consider the human rights implications of these new inclusions into the IHR in relation to equity and solidarity, medical countermeasures, core capacities, travel restrictions, vaccine certificates, social measures, accountability, and financing. We conclude with ideas for the future operation and reform of the IHR.

## 1. Bringing human rights into the IHR

The IHR have evolved to respond to the international challenges posed by infectious disease risks, turning to human rights as WHO governance came to see the linkages between public health prevention and human rights promotion.

### 1.1 Pre-2005

Arising out of a century of international health diplomacy for infectious disease prevention, WHO member states brought together these past international agreements in 1951 under the International Sanitary Regulations, which were renamed the International Health Regulations in 1969. The 1969 regime initially sought to bind all WHO member states to monitor outbreaks of specific named diseases (namely cholera, plague, and yellow fever). While providing an international framework for disease surveillance, this legal regime was narrow in scope, inadequate for state accountability, and inattentive to human rights.

Notwithstanding early IHR silence on human rights, the 1946 WHO Constitution recognized the right to health, as states worked in the United Nations (UN) to articulate the right to health and other health-related rights obligations under international human rights law, including obligations to “prevent, treat, and control” epidemic diseases under the 1966 *International Covenant on Economic Social and Cultural Rights* (ICESCR). With the UN focusing simultaneously on restrictions of civil and political rights to protect public health, states recognized in the 1966 *International Covenant on Civil and Political Rights* (ICCPR) that public health may be invoked as a basis for limiting certain rights such as freedom of movement, peaceful assembly and association where “necessary” and provided for by law. To elaborate the grounds for these limitations under the ICCPR, scholars developed the 1984 *Siracusa Principles on the Limitation and Derogation Provisions in the International Covenant on Civil and Political Rights*, which concluded that public health may be invoked as a ground for limiting certain rights to allow a state to take measures dealing with a serious threat to the health of the population.^6^

The Siracusa Principles recognized that such human rights restrictions should be undertaken only when (a) responding to a pressing public need, (b) necessary and proportionate to a legitimate aim, (c) prescribed by law and not imposed arbitrarily, and (d) applied as a last resort using the least restrictive means available. Calling for due regard to the IHR when restricting civil and political rights to protect public health, the Siracusa Principles would be increasingly applied — including by UN human rights bodies — to assess government measures in response to public health emergencies.^7^

Yet, with states increasingly unable to respond under the IHR to emerging and re-emerging infectious diseases in a rapidly globalizing world, the World Health Assembly (WHA) formally launched a WHO process in 1995 to broaden the scope of the treaty beyond cholera, plague, and yellow fever.^8^ The revision process progressed slowly until the emergence of Severe Acute Respiratory Syndrome (SARS), a new infectious disease threat not covered by the IHR. Limitations in effectively responding to this 2002–2003 outbreak underscored the inadequacy of the IHR, bringing new urgency to the revision process.^9^ Compounding this inadequacy, national governments had responded to SARS through public health actions that violate individual rights, resorting to sweeping isolation, quarantine, and surveillance measures that restricted individual liberties.^10^ These human rights violations in the SARS response raised an imperative to address human rights in IHR revisions.

### 1.2 IHR (2005)

As the impact of SARS mounted, WHO released the draft of a revised IHR, incorporating human rights into the IHR for the first time. States broadly recognized the importance of human rights in subsequent negotiations, and in May 2005, the WHA adopted the revised IHR (2005), codifying that states shall implement the IHR “with full respect for the dignity, human rights and fundamental freedoms of persons”.^11^ Drawing from the human rights balancing under Siracusa Principles, national measures under the IHR (article 43) must be based on scientific risk assessment and must not be more restrictive of international traffic, or more intrusive to individuals, than reasonably available alternatives.^12^

These IHR (2005) commitments introduced explicit human rights obligations under global health law, with additional rights-related provisions for non-invasive, least intrusive, and consensual health measures for travelers on arrival and departure (article 21), non-discriminatory implementation of the IHR (article 42), confidentiality of personal data (article 45), and requirements that travelers be treated with respect for their dignity and human rights when implementing health measures (article 32). The IHR in several provisions (including articles 17, 23, 31 and 43) requires its implementation to not be more invasive or intrusive to persons than reasonably available alternatives, wording which reflects the Siracusa Principles’ requirement that restrictions of rights not be more restrictive than required to achieve the purpose of the limitation.^13^ The IHR additionally gestures to extraterritorial human rights obligations of global solidarity (article 44), seeking international collaboration and assistance to support national public health capacities.^14^ These aspects of the revised IHR reflected a clear conceptual interconnection with state obligations under human rights law.^15^ Yet the IHR’s human rights provisions do not consider economic, social and cultural rights like health, education, food, work and social security, which may be significantly compromised during a pandemic.^16^

## 2. Human rights framing in the IHR amendment process

The limitations of the IHR’s provisions on human rights became all too apparent during the COVID pandemic, when responses entailed disproportionate and discriminatory limitations of a broader range of rights in the implementation of emergency measures including economic, social, and cultural rights (and especially the right to health).^17^ Gross disparities in global access to COVID-19 vaccines and other medical countermeasures have similarly illuminated the failure of states to heed the IHR’s obligations around capacity-building, solidarity, and cooperation in articles 5, 13, and 44 respectively.^18^ The wide spectrum of human rights repercussions in public health emergencies prompted the Independent Panel on Pandemic Preparedness and Response to commission a background paper on the theme, although its own analysis of human rights in its final report was lacking. These limitations also precipitated the development of the 2023 *Principles and Guidelines on Human Rights in Public Health Emergency Preparedness, Prevention, and Recovery*, which sought to address the lack of coherent guidance under international human rights law through clarifying the application of human rights to public health emergencies.^19^ These principles emphasize universality, equality, non-discrimination, and public participation, and outline positive obligations to mobilize resources and implement rights, including through the IHR.

In the wake of significant scrutiny of the IHR (2005) in the light of its functioning during the COVID-19 pandemic, in May 2022, the WHA formally initiated a revision process under the auspices of an Intergovernmental Working Group on Amendments to the IHR.^20^ Procedurally, under article 21 of the WHO Constitution, the WHA can agree on revisions to legally binding regulations without requiring ratification by national governments or domestic legislatures and with a lower approval threshold than treaties. All member states are required to comply with the enacted revised regulations except for those which raise rejections or reservations.

The Working Group invited member states to submit proposals and convened a Review Committee of independent experts to provide technical advice, as prescribed by the IHR.^21^ The proposed revisions, which were intended to be limited in scope, aimed to strengthen the IHR as an instrument for pandemic prevention, preparedness, and response through addressing equity, technological or other developments; improving implementation and compliance with the IHR; and assuring universal application to prevent the international spread of disease in an equitable manner.^22^ Whilst equity is mentioned in the proposals of amendments as a rationale for reform by states represented by the African Group, and other states such as India, Indonesia and Malaysia, the revision of the IHR was not originally initiated with the intention of increasing respect for and compliance with human rights obligations.^23^ Ultimately the amendment process was mostly state-led, with limited opportunity for civil society engagement, and without an independent human rights impact assessment or survey of conflicts between human rights and the proposals.^24^

The Review Committee issued a technical report in January 2023, but it only explicitly addressed human rights as an interpretive principle, and lacked any meaningful emphasis on human rights norms, principles, and specific obligations.^25^ For example, the report does not reflect the calls made by the UN Secretary General, WHO Director General, UN High Commissioner for Human Rights and UN human rights treaty bodies to center human rights in responses to the COVID-19 pandemic, including through protections for marginalized and vulnerable groups and greater accountability.^26^ The report merely underscores the importance of human rights in the revisions which produced the 2005 text, but without elaborating on the whole range of obligations that could be reflected or mainstreamed throughout the regulations.^27^ As an isolated comment, the Review Committee did welcome a proposal from the Africa Group to establish a requirement of “avoiding unnecessary interference with human rights” during public health measures, as such limitations “should only be imposed in accordance with the principles of legitimacy, necessity and proportionality, which means, inter alia, on a temporary basis and to the extent necessary”.^28^ Nonetheless, this proposal does not differ substantially from the status quo. By refraining from elaborating and mainstreaming human rights in the instrument, the risk is that the application of human rights obligations is left to each state’s interpretation.

Nevertheless, some states proposed amendments that consider solidarity and equity as well as the availability and affordability of care, concepts which resonate with comparable norms in human rights. For example, the African Group proposals markedly bear some parallel with the right to health obligation to assure available, accessible, acceptable, and good quality health care (AAAQ) as developed by the UN Committee on Economic, Social and Cultural Rights.^29^ These amendments introduce an allocation mechanism for PHEICs to ensure that resources are effectively available and affordable to all states. Moreover, the proposals reflect important elements of the right to health by suggesting a mandate that states regulate non-state actors in addition to state actors when it comes to observing WHO recommendations, participation in international allocation of resources, transparency in prices, and technology and know-how sharing.^30^

By contrast, other proposals, particularly from India, would have weakened the IHR’s human rights grounding by displacing the reference to “dignity, human rights, and fundamental freedoms of persons” as a fundamental pillar of the IHR.^31^ The Review Committee warned against this deletion, since the amendment would limit the interpretation of the IHR only to “equity, inclusivity, coherence, and in accordance with their common but differentiated responsibilities of the States Parties, taking into consideration their social and economic development.”^32^ Inasmuch as equity and human rights hold important complementarities (discussed further below), neither concept can adequately replace each other.

Hailed as a global health diplomacy achievement amidst a period of political instability, the IHR revisions were adopted on June 1, 2024 at the 77th WHA.^33^ Although not more explicit on human rights, the amendments were generally received as a timely advancement in global health governance — even though necessary reforms were left to be negotiated through a future Pandemic Agreement (including stronger rules on pathogen access and vaccine distribution).^34^ Notable highlights of the amendments include conceptual improvements, such as the definition of “pandemic” in international law as a new category of PHEIC (Articles 1 and 12), the inclusion of “preparedness” within the scope of the IHR and core capacities (e.g. Article 2), and the promotion of equity and solidarity alongside human rights as overarching principles (Article 3).

In a significant shift, efforts to enhance compliance and implementation were also reflected in several amendments, including the establishment of a State Parties’ Committee for Implementation of the IHR (Article 54 bis) and the creation of National IHR Authorities (Article 4), responsible for ensuring local implementation. In other areas, the reforms addressed critical issues in financing and access to countermeasures, albeit with open-ended commitments that lack well-defined outcomes, in particular to reduce global health disparities. The revised IHR pave the way for further deliberations and engagement on these matters, including the creation of the Coordinating Financial Mechanism for the implementation of the IHR (Article 44) and the competence of the WHO Director to “facilitate, and work to remove barriers to, timely and equitable access by states to relevant health products” (Article 13.8).

## 3. Key human rights issues for the amended IHR

It is essential to assess possible synergies and tensions between the reforms and human rights obligations to support the implementation of the IHR in ways that advance, rather than undermine, existing state obligations under international human rights law. In the following section we consider how (and whether) key human rights issues were addressed in IHR reforms and achieve this goal. We highlight that while some revisions were positive, including the inclusion of equity and solidarity as grounding principles, as well as provisions on medical countermeasures, core capacities, accountability and financing, they also represent missed opportunities for explicit protection of human rights in line with international human rights law standards. Moreover, the revisions are notable for what they didn’t do and for stopping short of addressing other measures with human rights implications, such as travel restrictions, vaccine certificates and social measures.

### 3.1 Equity, solidarity, and human rights

From a human rights perspective, perhaps the most conceptually significant inclusion in the 2024 reforms is the addition of equity and solidarity to IHR article 3. Article 3.1 now states that the “implementation of these Regulations shall be with full respect for the dignity, human rights and fundamental freedoms of persons, and shall promote equity and solidarity.” These insertions reflect advocacy during the drafting process in which equity emerged as a significant underlying principle within the IHR final proposed text, being cited ten times in the document, compared to just two references to human rights.^35^ These are normatively important inclusions that arguably hold the potential to fundamentally reshape the agreement, particularly insofar as gross inequities like access to essential pandemic health goods are concerned. At the same time, the content of these terms is not defined in the IHR amendments. Nor are these terms linked to comparable norms in international human rights law.

Despite the increased role of equity in international law and calls for its expansion in global health governance, the IHR’s embrace of equity does not fully align with a rights-based approach.^36^ International courts and tribunals have generally regarded equity as part of customary international law or a general principle of international law in inter-state disputes.^37^ Yet equity increasingly is used in international law to frame the treatment of individuals and groups; for example the concept of “intergenerational equity” has been articulated in treaties under international climate law.^38^ Within global health, equity has been a framing concept to address systematic differences in health status between different population groups at the national level and at the global level, including in calls for vaccine equity during the COVID-19 pandemic. Yet despite normative overlaps between equity and rights to equality and non-discrimination, the relationship between equity in an amended IHR and these and other human rights norms is far from clear.

Certainly, the conceptions of justice and distributive outcomes inherent in the concepts of equity and solidarity are valuable. They are complementary to human rights frameworks that emphasize substantive and not merely formal equality. At the same time, human rights obligations and principles are conceptualized and invoked at an individual and group level (in contrast to inter-state relations), providing these individuals and groups crucial protection against the harms caused by inequitable and overly invasive public health measures.^39^ Human rights operate not only in substantive outcomes including specific and detailed freedoms and entitlements and in terms of the distribution of resources, but also in procedural aspects and minimum guarantees of deliberations (such as transparent, accountable, and participatory processes; and access to justice). These substantive and procedural norms and standards could offer concrete guidance to the operationalization of equity in the IHR.

### 3.2. Access to medical countermeasures

While the 2005 IHR aimed to coordinate and develop the ability to respond to infectious disease, it was silent on duties to ensure access to medical countermeasures during a pandemic. Indeed, the term “medical countermeasures” does not appear in the IHR (2005) itself and was subsequently developed by governments to designate medicines, vaccines, and antitoxins deemed necessary to protect national security in the context of biological threats.^40^ The WHO subsequently integrated a broader conception of medical countermeasures beyond security threats into benchmarks for IHR capacities measuring the development of systems to activate and coordinate such measures during a public health emergency.^41^

The inadequacy of the IHR’s approach to medical countermeasures was dramatically illustrated during COVID-19 as stark global disparities in access to COVID-19 vaccines quickly emerged, a situation that was met with widespread concern by many international human rights bodies.^42^ These weaknesses are outlined in the IHR Review Committee’s final report, which acknowledges that the IHR were not designed to address more pervasive issues exposed by COVID-19, for example, the persistent and gross disparities in access to life-saving medical countermeasures, including not only diagnostics, personal protective equipment, treatments, medical devices and vaccines, but also the commodities necessary for the manufacturing, packaging and distribution of such countermeasures.^43^

The report proposes amending IHR articles 15, 16, and 17 to allow the WHO Director General to issue temporary and standing recommendations on access to “health products, technologies and know-how, including an allocation mechanism for their fair and equitable access”.^44^ When it came to IHR article 44 on collaboration and assistance, the Committee broadly supported the principles of equitable access to countermeasures but questioned whether this issue fell within the scope of the IHR.^45^

The amended IHR adds several new provisions that resoundingly resolve the question of whether this issue falls within the scope of the IHR, even as they impose relatively “soft” corresponding obligations on the WHO and IHR States Parties. A definition of “relevant health products” is added to the IHR, as:

those health products needed to respond to public health emergencies of international concern, including pandemic emergencies, which may include medicines, vaccines, diagnostics, medical devices, vector control products, personal protective equipment, decontamination products, assistive products, antidotes, cell- and gene-based therapies, and other health technologies.^46^

Several other articles are amended to recognize WHO and state party responsibilities to ensure equitable access to health products as a core component and criteria of a public health response under the IHR. For example, article 13 on public health responses is extensively amended to recognize both WHO and state party duties to assure “equitable access to relevant health products.” The WHO undertakes to “facilitate, and work to remove barriers to timely and equitable access by States Parties to relevant health products … based on public health risks and needs.”^47^ The WHO Director-General undertakes to address in their recommendations public health needs and availability, accessibility, and affordability of health products (article 13.8.a), to use WHO and/or State mechanisms and networks to facilitate timely and equitable access to relevant health products based on public health needs (article 13.8.b); and to support states on request to scale up and geographically diversify the production of relevant health products (article 13.8.c); to share product dossiers for health products to states with the manufacturer’s consent (article 13.8.d); and to support states to promote research and development and strengthen local production of quality, safe and effective relevant health products (article 13.8.e). The WHO Director-General is also authorized to extend temporary and standing recommendations to address health measures in relation to relevant health products,^48^ and to consider as a criterion for such recommendations the availability and accessibility of relevant health products.^49^

Nowhere does the IHR expressly recognize a right to access countermeasures (including essential vaccines and antiviral medicines) that places clear and strong obligations on states to ensure fair distribution and equitable access to such health goods including through regulating non-state actors. And nowhere are measures to mitigate the impact of trade-related intellectual property rights on equitable access to medical countermeasures acknowledged in the IHR, including TRIPS flexibilities and waivers of intellectual property rights.

State duties in this regard are considerably vaguer and weaker: IHR state parties must “collaborate with, and assist each other and to support WHO-coordinated response activities,” including through supporting WHO and engaging with domestic stakeholders to facilitate equitable access to relevant health products for responding to a PHEIC including a pandemic emergency, and making available, “as appropriate, relevant terms of their research and development agreements for relevant health products related to promoting equitable access to such products during a public health emergency of international concern, including a pandemic emergency.”^50^

The novel insertion into the IHR of duties around equitable access to affordable health products is unquestionably a sea change, and a remedy to the former silence of the IHR on this question. Yet as in the rest of the IHR, these amendments do not recognize or link to comparable rights in international human rights law, a puzzling elision given that equitable access to affordable medical countermeasures during a pandemic is a fundamental entitlement under the ICESCR.^51^ For example, the UN Committee on Economic, Social and Cultural Rights has reinforced that access to medicines are core obligations and essential elements of ICESCR rights to health and science.^52^ Accordingly, states hold human rights obligations to assure access to affordable essential medicines,^53^ and to “prevent unreasonably high costs for access to essential medicines … from undermining the rights of large segments of the population to health”,^54^ including through using Agreement on Trade-Related Aspects of Intellectual Property Rights (TRIPS) flexibilities.^55^ The Committee urged states to support the waiver of TRIPS to assure the global affordability of vaccines and has framed the inequitable distribution of COVID-19 vaccines as contrary “to the extraterritorial obligations of States.”^56^

While the reforms never acknowledge the human rights dimensions of access to medicines and vaccines, they implicitly reflect some of the key conceptual dimensions of this right when it comes to accessibility and affordability. Yet by framing access as a question of equity instead of equality or the right to health, these duties remain delinked from the specific duties of this body of law. Nowhere does the IHR expressly recognize a right to access countermeasures (including essential vaccines and antiviral medicines) that places clear and strong obligations on states to ensure fair distribution and equitable access to such health goods including through regulating non-state actors. And nowhere are measures to mitigate the impact of trade-related intellectual property rights on equitable access to medical countermeasures acknowledged in the IHR, including TRIPS flexibilities and waivers of intellectual property rights. These elisions are unsurprising given the contested history and present of policy and law relating to global access to medicines. Yet grossly disparate vaccine access during COVID-19 underscores how important it is that states hold strong and binding legal obligations that appropriately balance efforts to assure access to domestic populations in ways that do not compromise access in other (and especially lower-resourced) countries and populations.

### 3.3 Core capacities

Articles 5 and 13 of the 2005 IHR required states to develop, strengthen, and maintain the capacity to detect, assess, notify, report, and respond to public health risks and emergencies of international concern. A set of “core capacities” under these articles, which should be implemented within five years, was listed in Annex 1, and unpacked in state reporting guidelines.^57^ These capacities were grouped in two areas: a surveillance and response system including public health infrastructure, staffing, reporting, communication, and a national public health emergency response plan; and measures at points of entry and exit to control international spread. Prior to the COVID-19 pandemic, many states had failed to implement core capacities (by 2015 two-thirds of states had already fallen short of compliance with existing core capacities)^58^ which has been attributed to a combination of a mismatch with public health capacities in some states, competing public health priorities, limited international support in this area, and weak international oversight.^59^ The chaotic response by many states to the COVID-19 outbreak further highlighted limitations in preparedness by states in areas already covered by core capacity requirements; preparedness in other areas critical for a pandemic response, including medical products and the health service, with many countries lacking necessary products and health service capabilities; and in coordination and solidarity between states as seen in nationalist responses undermining an equitable response worldwide.^60^

Accordingly, the 2024 amendments broadened surveillance (article 5) and response (article 13) obligations to new obligations on prevention and a more holistic set of preparedness obligations, requiring states to develop capacities to “prevent, detect, assess, notify and report events” (article 5) and to “prevent, prepare for, and respond promptly and effectively to public health risks and public health emergencies of international concern, including a pandemic emergency, including in fragile and humanitarian settings.” Correspondingly, Annex 1 builds on previous narrower requirements for surveillance and response under the 2005 IHR to embrace a broader set of core capacities including preparing for the implementation, and immediately implementing, preliminary control measures; preparing to provide and facilitate access to relevant health services and products; logistics; and risk communication including addressing misinformation and disinformation. States must also engage with relevant stakeholders including local communities in preparing for and responding to a response. A new onus is placed on international collaboration in the light of IHR Article 44 to enable states to develop, strengthen and maintain core capacities.

Core capacities, including the 2024 amendments, resonate with states’ right to health obligations to take measures to prevent, treat, and control epidemic diseases under ICESCR article 12.2.c. Analyzing links between core capacities under the IHR (2005) and right to health “core obligations,” which had hitherto received limited analysis in relation to the threat of PHEICs, Toebes, Forman, and Bartolini have also pointed to normative synergy between IHR core capacities and right to health “core obligations’”^61^ (which are obligations which all states must prioritize); in particular they argue that the obligation to “adopt a national health strategy and plan of action to address the population’s health concerns,” should be interpreted in the light of IHR core capacities, considering the risks of PHEIC to the right to health. The revisions to core capacities in 2024 have entailed further alignments with human rights including: the focus of right to health core obligations on the accessibility of healthcare, a role for participatory processes, and the importance of obligations of international cooperation to ensure necessary pandemic preparations and response.

Despite such synergies, there are also potential tensions between core capacities and right to health obligations which may be exacerbated by proposed amendments. Experience during the COVID-19 pandemic highlighted that control measures are often designed and implemented in ways that violate human rights, and access to health services and medical products often fall short of human rights requirements of equality and non-discrimination. Although human rights and equity are now cross-cutting principles in the IHR, it is a shortcoming of the IHR reforms that these principles are not addressed more explicitly to reinforce human rights-compliant PHEIC preparations including through reducing the very real risks to dignity, well-being, and livelihoods of non-human rights based PHEIC and pandemic responses.

Furthermore, while it is welcome that the IHR core capacities recognize duties to provide health services “necessary for responding to public health risks and events,” they do not address broader questions of health system strengthening and universal health coverage, both of which are recognized as important aspects of PHEIC prevention and preparedness as well as right to health obligations.^62^ Nor do they specifically elaborate on preparing to maintain other health services and public health interventions during a PHEIC or pandemic response. During the COVID-19 pandemic, human rights mechanisms and scholars highlighted concerns about the parlous state of many health systems after years of neglect, as well as about the restrictions of other health services and determinants of health like adequate food or social security in pandemic responses, often resulting in inequalities and discrimination in access to care and determinants of health.^63^ Yet it is notable that the IHR Review Committee was cautious about introducing equitable financing mechanisms and health system strengthening as part of the IHR, noting that this would fall outside the focus of the IHR and the WHO mandate in this area and would require more detailed regulation more broadly. It is arguable that the Committee’s reluctance to address these broader health systems dimensions within the IHR also reflects its resistance to integrating right to health duties more fully into the IHR.

Furthermore, some suggested amendments raise issues of technical and financial feasibility, particularly for low- and middle-income countries. While core capacities are framed as technical measures, there is limited acknowledgement of historic and current structural inequalities that act as barriers to the public health systems that underpin core capacities in the Global South. Broadening of core capacities to include healthcare and healthcare products are examples of unfunded mandates that will significantly challenge many states. Although collaboration is highlighted as an obligation in relation to core capacities, the mechanisms to achieve this in practice remain elusive.

Fulfilling core capacity measures should ensure that health policy be formulated to consider the health needs of the population as established by scientific evidence, national and local priorities as determined under democratic processes,^64^ as well as the participation of affected populations in health decision-making. Moreover, the precise legal and normative relationship of human rights and IHR obligations remains opaque and under-operationalized given limited interaction between the human rights and global health regimes. For example, there remains a lack of clarity about the bearing of human rights under IHR article 3 on core capacities. Explicit reference and incorporation of human rights norms and obligations should have been a part of the drafting process of the IHR revisions, including core obligations and obligations related to the health system more generally. Doing so would have supported an integrated rather than fragmented set of obligations across international legal regimes more conducive to protecting both public health and human rights.

### 3.4 Travel restrictions

In responding to the COVID-19 pandemic, many states ignored public health interventions recommended by WHO in a rush to implement selective bans on international travel. The IHR’s article 43 requires that state responses “shall not be more restrictive of international traffic and not more invasive or intrusive to persons than reasonably available alternatives.” However, government responses, undertaken at times for domestic political reasons and without adequate public health evidence, divided the world when global solidarity was needed most. Despite continued WHO opposition to such travel restrictions, states continued to impose discriminatory travel restrictions reflexively, prioritizing reactionary measures without offering public health justifications.^65^

These travel restrictions undermined respect for human rights in the IHR. Instead of the IHR being implemented “with full respect for the dignity, human rights and fundamental freedoms of persons,”^66^ travel bans restricted the human right to freedom of movement, blocked the international assistance needed in the pandemic response,^67^ and limited a range of other civil, political, economic, and social rights. Such unnecessary (and often discriminatory) travel bans undermined global solidarity, driving nations apart through economic isolation and rights violations. The IHR cannot be effective where governments pursue travel restrictions without public health evidence, restricting individual movements, economic activities, and humanitarian responses in ways that contravene WHO guidance, stoke nationalist and racist discrimination, and violate human rights.^68^

Accordingly, it is imperative to understand when travel restrictions are necessary and proportionate under the IHR, and to align global health law and human rights law obligations. Despite (or perhaps because of) the contentious nature of travel restrictions, the 2024 reforms did little to address these outside of adding a provision to the IHR that allows impacted states to request consultations with the implementing state party or through the Director-General.^69^ Such consultations must be confidential and for the purpose of clarifying “the scientific information and public health rationale underlying the measure and to find a mutually acceptable solution.” While this addition to article 43 could help improve the transparency of travel restrictions, it is doubtful that state consultations will resolve the larger problem of discriminatory travel restrictions witnessed during COVID-19.

These gaps illustrate that the IHR will need to be further amended to provide greater specificity in implementing travel restrictions and border closures that are appropriate, non-discriminatory, uphold transparent processes, ensure evidence-based practices, respect human rights protections, and strengthen WHO recommendations. Specifically, such reforms must ensure that travel restrictions conform with both human rights and IHR principles of health necessity and proportionality and are accompanied by the correlative IHR and human rights duties of international collaboration and assistance rooted substantively in global solidarity.^70^

The vesting of authority in the WHO to authorize such measures in relation to forthcoming emergencies requires it to consider such human rights questions in its decision-making in accordance with IHR article 3 and its constitutional mandate. Yet this imperative is not explicitly codified in the current IHR and the IHR reforms do not explicitly recognize WHO’s authority in this regard.

### 3.5 Vaccine certificates

The 2005 IHR has recognized only limited permissible use of vaccination certificates. For instance, it provides that states parties may require proof of yellow fever vaccination or prophylaxis for travelers as a condition of entry (Annex 7). Beyond this, in line with its general purpose of preventing and controlling the international spread of disease whilst minimizing interference with international traffic and trade (article 2), the IHR prohibits states from requiring health documents for international traffic for other conditions, except in circumstances where the WHO has issued such recommendations (article 35). In cases where vaccines are foreseen, the 2005 IHR provides that entry should not be denied for persons in possession of certificates of vaccination or other prophylaxis (article 36).

Following the rollout of the first COVID-19 vaccines, many states adopted vaccine requirements for entry and exit. In addition to specific vaccine certificates, they also introduced other health documentation and travel requirements, such as negative antigen or PCR test certificates prior to or after travel; regulations around paper and digital certification; and quarantine or isolation requirements. Even within these requirements, amongst other restrictions, some states refused to recognize tests administered in countries in the Global South. Additionally, vaccination certificates as a condition for international travel is in contravention of WHO guidance to refrain from such practices^71^ and of states’ IHR obligations. Vaccination alone came to be recognized as ineffective in preventing COVID-19 transmission, while certification for travel restricted rights to freedom of movement, privacy and family life,^72^ raising concerns around necessity and proportionality.^73^

Like travel bans, international travel vaccine certificate requirements disadvantaged populations in the Global South who remained at the back of the vaccine rollout queue. These disparities compounded the injustice of global vaccine apartheid as Global North states bought up vaccine stock, blocked proposals for intellectual property waivers on COVID-19 vaccines under the TRIPS Agreement and failed to uphold pledges for equitable vaccine distribution under the COVAX scheme. Digitalization requirements further raised concerns about equity in the context of the digital divide between high- and low- and middle-income countries as well as within countries. Global vaccine disparities illustrated an interplay of power relations between countries, global racialized health and rights inequalities, security concerns reminiscent of the era of colonial health,^74^ ^,^ and commercial priorities strongly redolent of the ongoing neoliberal era.

States proposed an article 36 amendment to recognize other types of proofs and certificates that would attest to a decreased public health risk “particularly where a vaccine or prophylaxis has not yet been made available for a disease in respect of which a public health emergency of international concern has been declared.”^75^ Studies undertaken during the COVID-19 pandemic suggested that other types of testing and certification (e.g. exit screening test and certificates), and entry screening together with isolation guidance may help protect the right to health through limiting exposure for other travelers and contacts, although they may have a limited impact in slowing the spread of disease^76^

The vesting of authority in the WHO to authorize such measures in relation to forthcoming emergencies requires it to consider such human rights questions in its decision-making in accordance with IHR article 3 and its constitutional mandate. Yet this imperative is not explicitly codified in the current IHR and the IHR reforms do not explicitly recognize WHO’s authority in this regard.

A second area of suggested revisions concerned digitalization of certification, interoperability and data protection, with assistance envisaged for low- and middle-income countries to develop such systems. While pragmatic and largely in line with existing WHO-supported processes, the Review Committee raised concerns about inequalities given digital divides between countries and different population groups.^77^ Yet these suggested revisions were not adopted in ways that reduce risks of digital exclusion, with IHR (2024) article 35 recognizing that documents may be issued in either digital or non-digital format, subject to other international agreements.

### 3.6 Social measures

Poverty, discrimination, and inequality are both determinants of risk and outcomes of epidemics and pandemics such as Ebola, Zika and COVID-19. With devastating impacts on health systems, food security, social security, education, and employment, international human rights bodies have highlighted that states must guarantee economic, social, and cultural rights in public health emergency responses and recovery, including for marginalized populations.^78^ Yet the purpose of the IHR is focused narrowly on protecting public health through technical and management measures that prevent and control the international spread of diseases and do not interfere with international trade.^79^ The IHR does not overtly extend to addressing individual or systemic economic and social vulnerabilities to infectious diseases or to regulating states’ socio-economic responses. Though the IHR requires states to ensure human rights and non-discrimination in implementing the Regulations, the overriding focus has been on civil and political rights such as rights to freedom of movement, privacy, and peaceful assembly, and indeed the drafting of the IHR was conceived particularly with such rights in mind.^80^ However, Article 3 protections can and should also be interpreted to extend to avoiding interference with economic, social and cultural rights, particularly the right to health which is centrally implicated in multiple dimensions of the IHR.

It is certainly positive that IHR reforms advance in a small way towards recognizing socioeconomic risks in pandemics and the rights associated with them. For example, the definition of a pandemic emergency includes “social and economic disruption,” and newly enshrined principles such as equity and solidarity have a socioeconomic resonance. Equitable access to health products reflects comparable right to health norms regarding accessible and affordable medicines and vaccines, and the new duties in core capacities in terms of healthcare preparedness and access reflect broader right to health concerns. Yet beyond these advancements, the regulations do not overtly shift away from its technical approach and civil and political rights focus in its operative articles.

Notably, the IHR is inadequate when it comes to positive obligations for states to build robust health systems accessible to all and resilient to public health emergencies.^81^ Such shortcomings drove calls for a new approach to global governance of public health emergencies under a new pandemic instrument that would address preparedness and response gaps in the IHR.^82^

### 3.7 Accountability of the IHR in relation to human rights

Significantly, extant compliance and accountability mechanisms have been inadequate within the IHR. The 2005 IHR prioritized negotiation between state parties if there is a dispute, referral of the dispute to the Director General, arbitration, and resorting to the dispute settlement mechanisms of other intergovernmental organizations. Additionally, in article 56, the Regulations provide “in the event of a dispute between WHO and one or more States Parties concerning the interpretation or application of these Regulations, the matter shall be submitted to the Health Assembly.” The current system is underutilized and too state-centric. The need for a more effective dispute settlement mechanism within the regime could not be more apparent. This is a ripe area for future reforms.

In attempting to overcome some of these deficiencies, the 2024 revisions were aimed at enhancing accountability primarily in terms of monitoring and implementing the regulations themselves, rather than addressing human rights violations that may arise from their application. These revisions explicitly focused on improving assistance, cooperation, and financing aspects of the regulations (article 54.1), and do not offer a specific mechanism for scrutinizing state measures or compliance with recommendations during a PHEIC. The States Parties Committee for the Implementation of the IHR is also not formulated as a robust monitoring body; instead it is limited to a consultative role, operating in a non-adversarial, non-punitive, and transparent manner (article 54), with at least a meeting every two years (article 54.2). As a result, it remains unclear how the Committee’s procedures will impact or regulate state behavior (its terms of reference were left to be defined), or if their mandate will closely review interstate inequities and human rights obligations, particularly concerning international cooperation and assistance.^83^ Similarly, the new National IHR Authority that states are to create is only described as “an entity designated or established by the State Party at the national level to coordinate the implementation of these Regulations within the jurisdiction of the State Party,” with no requirements to review the human rights implications of IHR implementation.

Notwithstanding the current reforms, the procedures and framework that are set out in the Regulations provide only the broad parameters for emergency decision-making. The procedures do not explicitly require an assessment of what the human rights consequences of temporary or standing recommendations might be as part of the WHO’s decision-making. Further, in article 48.1, the Regulations require the Director General to consult with the committee of experts before deciding whether to terminate an emergency or to modify a previously issued recommendation. However, considering the human rights implications of terminating an emergency or modifying a previously issued recommendation is not required. Moreover, while IHR articles 50–53 state that the WHO’s emergency response is subject to review by an expert committee that can issue a non-binding report, nothing currently requires a human rights evaluation of the WHO’s response or consideration of whether its recommendations violate human rights.

Additionally, according to IHR article 1, the WHO’s temporary and standing recommendations to states are non-binding. Under article 43.1.a.b of the Regulations, state parties are permitted to implement health measures in response to a PHEIC that “achieve[s] the same or greater level of health protection than WHO recommendations.” States that decide to adopt such measures are required under IHR article 43.5 to “provide to WHO the public health rationale and relevant scientific information for it.” IHR article 43.6 requires parties that apply additional measures that significantly interfere, to within three months undertake a review “taking into account the advice of WHO.” However, as witnessed with the COVID-19 pandemic as well as with the Ebola epidemic, states have failed to comply with these provisions.^84^

The consequences of the lack of a robust compliance mechanism for assessing when countries deviate from the WHO’s recommendations allow significant room for countries to implement policies not based on any public health rationale. As elaborated above with the COVID-19 pandemic, the lack of any compliance mechanism also allows for the violation of human rights. Indeed, the WHO’s inability to impose sanctions on state parties in the event of noncompliance with its recommendations also means that potentially protective human rights provisions within the IHR, like article 42 that require state parties to implement and apply health measures “in a transparent and non-discriminatory manner,” lacks much enforceability and is insufficiently protective of human rights. The WHO has remarked that perhaps “the best incentives for compliance are ‘peer pressure’ and public knowledge” since “[s]tates do not want to be isolated.”^85^ Yet often it is the WHO that is isolated as states sideline the organization and enact policies contrary to public health and human rights.

At the same time, for human rights to be better respected in the IHR, we need institutional linkages between the IHR and the international human rights system. Such linkages could include measures that link state compliance under the IHR with the UN human rights regime, such as state reporting under the UN Committee on Economic, Social and Cultural Rights, the Universal Periodic Review mechanism, and UN Special Rapporteurs and Independent Experts on human rights and health. In addition, as with other human rights accountability mechanisms, individuals, civil society, and other actors need to be allowed to bring disputes and claims involving human rights violations and have these claims considered, investigated, and where merited, addressed.

### 3.8 Financing and human rights

In 2024, IHR article 44 concerning collaboration and assistance was extensively amended to include novel duties around financing aimed at improving IHR implementation especially for low- and middle-income countries. States agree “to collaborate with each other to the extent possible” and to mobilize financial resources to facilitate IHR implementation especially for low- and middle-income countries;^86^ to “maintain or increase domestic funding” and to collaborate through international cooperation and assistance to strengthen sustainable financing for IHR implementation.^87^ These financing duties extend to facilitating access to health products, with the WHO required to collaborate with and assist States Parties to facilitate access to relevant health products.^88^

In addition, a new coordinating financial mechanism is anticipated under the 2024 reforms to promote “timely, predictable, and sustainable financing” to implement the IHR and “develop, strengthen, and maintain core capacities;”^89^ and to maximize existing financing and mobilized new financing for more effective implementation needs that meet state priorities, especially those of developing countries.^90^ These provisions acknowledge that the need to improve the IHR’s efficacy extends to greater financial support for low- and middle-income countries and create duties and mechanisms in this regard. Yet while these duties suggest extraterritorial human rights duties to cooperate and assist low- and middle-income countries to realize human rights, again there are no explicit linkages to these duties when there should be.

## 4. Conclusion

The COVID-19 pandemic illustrates that the lives of millions are threatened by infectious diseases and that effective global health law is crucial to mitigating these risks and harms. The IHR did not effectively respond to the threats and harms of COVID-19, given the significant gaps in the instrument and inadequate mechanisms for accountability that our analysis has surfaced. The reforms to the IHR offered an opportunity to better meet these pressing needs and to mount a more effective public health response.

The IHR amendments on equity, solidarity, access to vaccines, financing, and pandemic preparedness are welcome and positive additions to this instrument. Yet government duties during a pandemic extend far beyond the IHR and considerations of trade or security. State duties to address pandemics are required under human rights law, rooted in fundamental rights such as the right to health, science, life, and equality, amongst other rights. Greater attention to human rights in the IHR could have allowed for systemic integration between human rights and global health law in ways that serve public health and human rights, rather than causing further fragmentation and incoherence in these bodies of law. The threat of a lack of linkage is that a vital opportunity to assist states in fulfilling their pandemic-related human rights obligations was missed, which bodes poorly for the successful national and global governance of future disease threats. Moreover, greater inclusion of and linkages to human rights norms and institutions in the 2024 IHR revisions could have offered a means of assuring a greater balance between global public health and security and commercial concerns, and of protecting against the kinds of gross disparities and inequities we saw with COVID-19.

The opportunity for reform opened by the pandemic will not come again soon and the pandemic instrument in negotiation is unlikely to fill these gaps. Inasmuch as many of the 2024 reforms will likely bolster the efficacy of the IHR in important ways, a key opportunity for the IHR to better protect human rights has been lost. In future pandemics, the IHR will more than likely continue to fail to adequately protect human rights, and with that, public and global health. It will fall, as always, to civil society and human rights advocates globally to push states and other actors to remediate the shortcomings of the IHR when it comes to protecting health-related human rights during public health crises and beyond.

## Note

Lisa Forman is supported by the Connaught Global Challenge Award under Grant 512634 and the Canada Research Chair Fund under Grant 497890.
